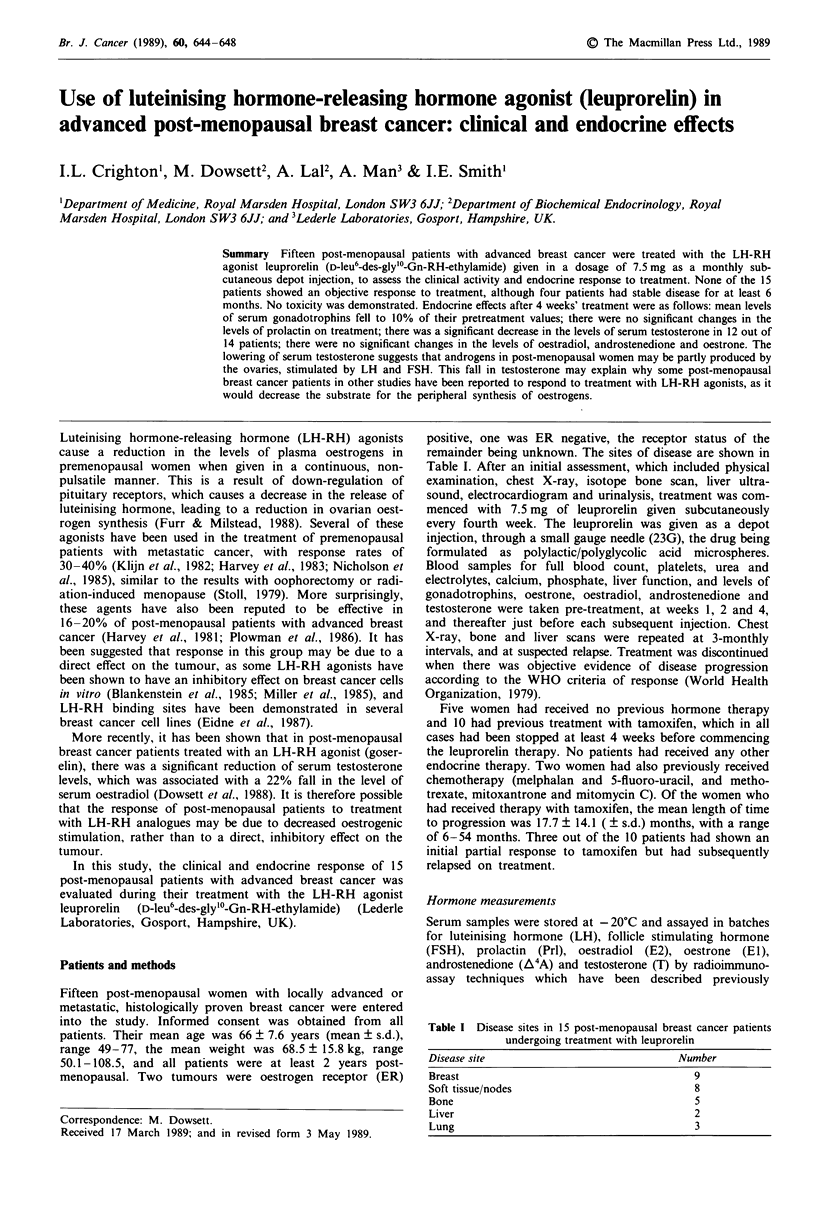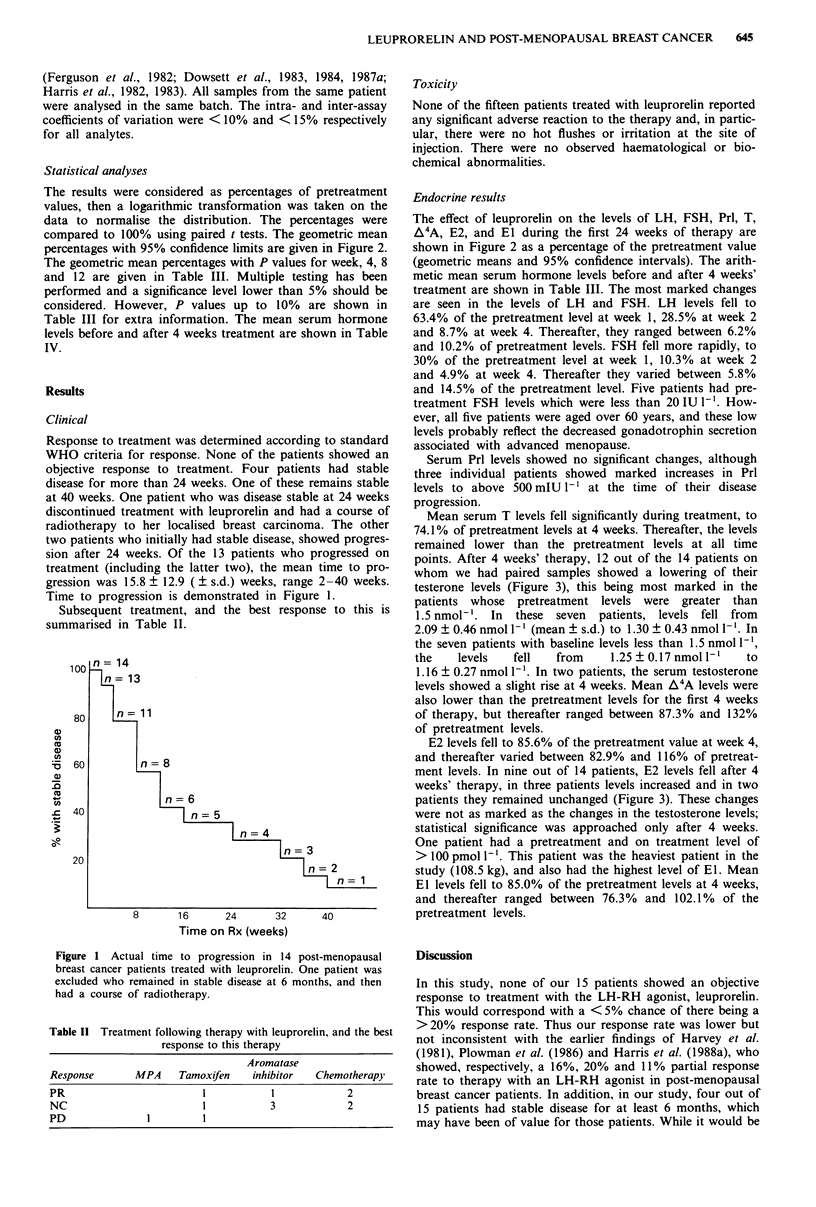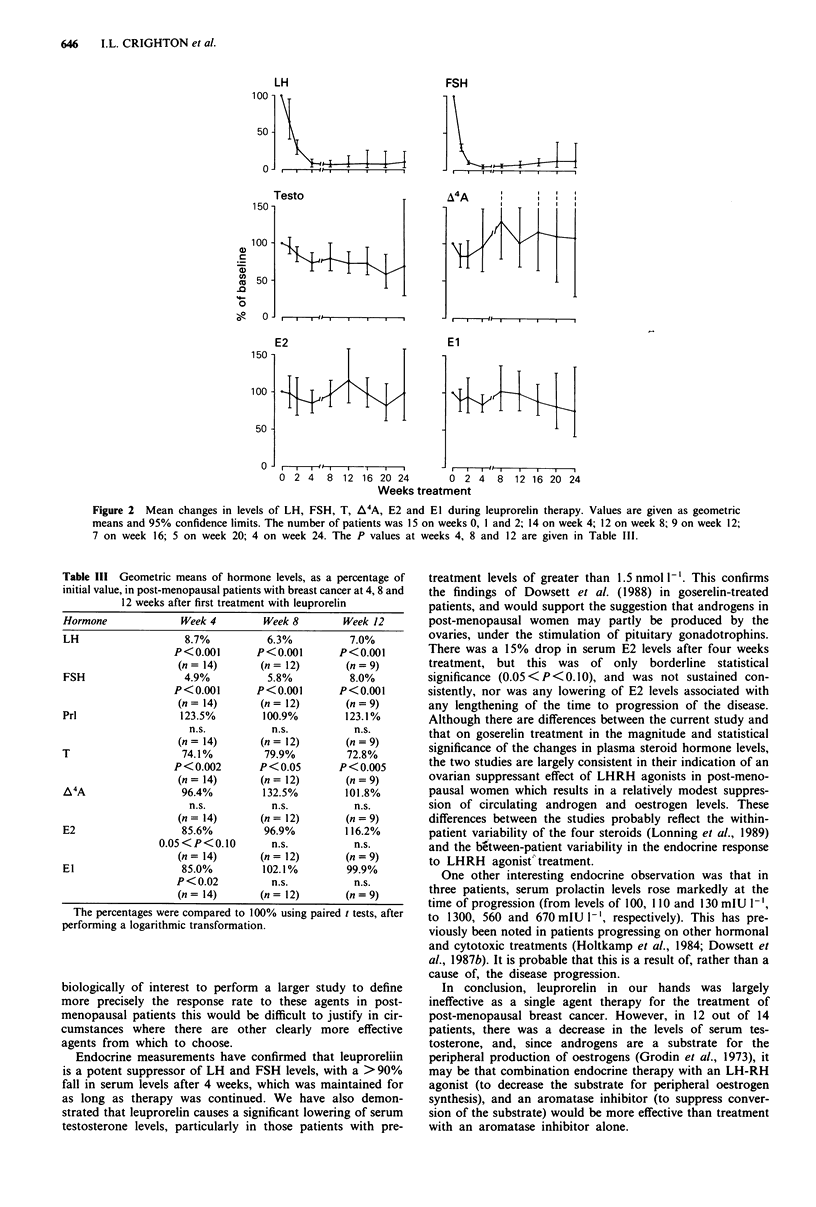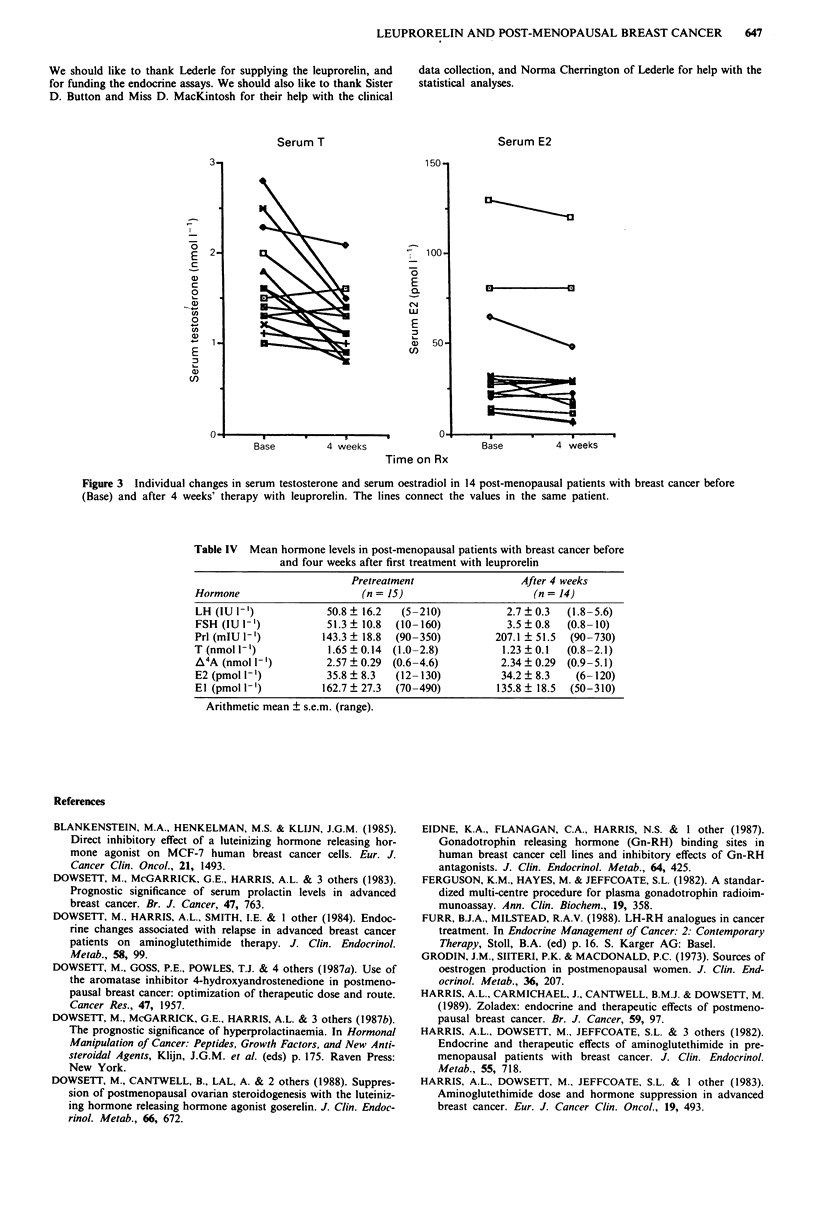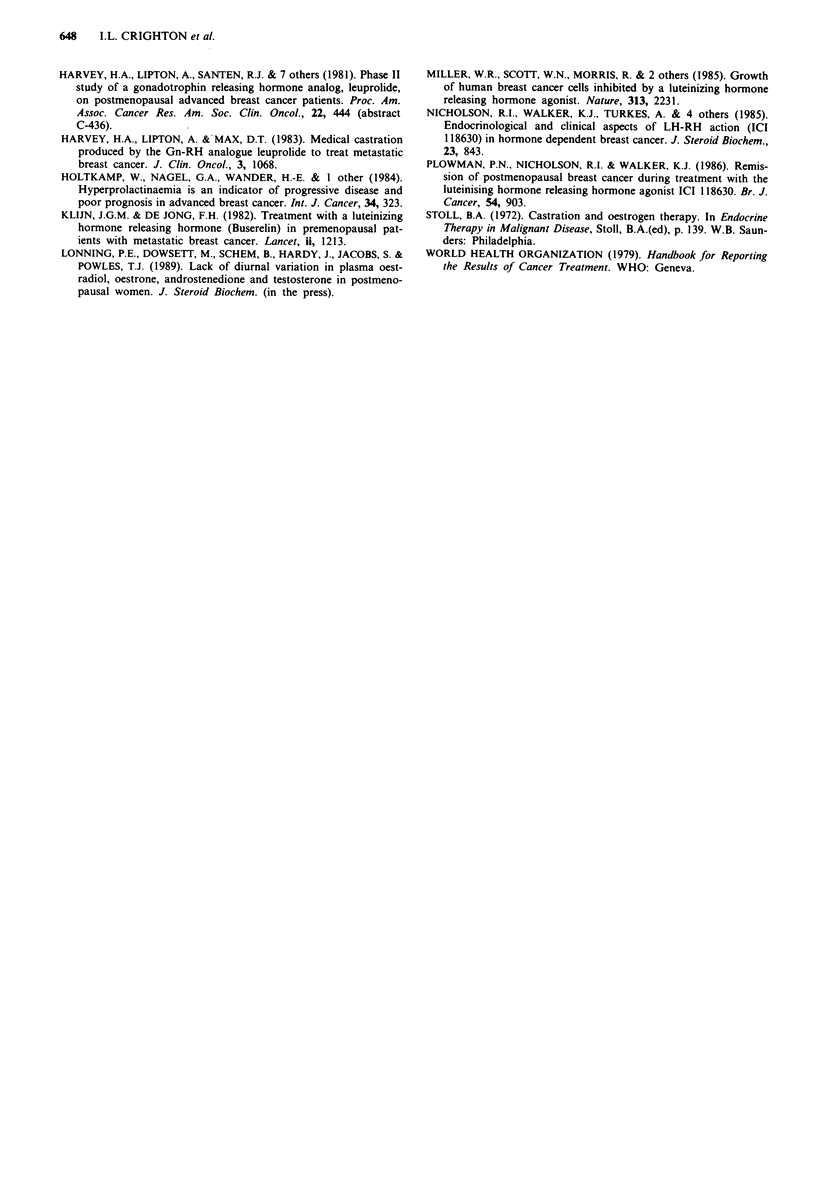# Use of luteinising hormone-releasing hormone agonist (leuprorelin) in advanced post-menopausal breast cancer: clinical and endocrine effects.

**DOI:** 10.1038/bjc.1989.331

**Published:** 1989-10

**Authors:** I. L. Crighton, M. Dowsett, A. Lal, A. Man, I. E. Smith

**Affiliations:** Department of Medicine, Royal Marsden Hospital, London, UK.

## Abstract

Fifteen post-menopausal patients with advanced breast cancer were treated with the LH-RH agonist leuprorelin (D-leu6-des-gly10-Gn-RH-ethylamide) given in a dosage of 7.5 mg as a monthly subcutaneous depot injection, to assess the clinical activity and endocrine response to treatment. None of the 15 patients showed an objective response to treatment, although four patients had stable disease for at least 6 months. No toxicity was demonstrated. Endocrine effects after 4 weeks' treatment were as follows: mean levels of serum gonadotrophins fell to 10% of their pretreatment values; there were no significant changes in the levels of prolactin on treatment; there was a significant decrease in the levels of serum testosterone in 12 out of 14 patients; there were no significant changes in the levels of oestradiol, androstenedione and oestrone. The lowering of serum testosterone suggests that androgens in post-menopausal women may be partly produced by the ovaries, stimulated by LH and FSH. This fall in testosterone may explain why some post-menopausal breast cancer patients in other studies have been reported to respond to treatment with LH-RH agonists, as it would decrease the substrate for the peripheral synthesis of oestrogens.


Br. J. Cancer (1989), 60, 644–648 © The Macmillan Press Ltd., 1989

### Use of luteinising hormone-releasing hormone agonist (leuprorelin) in advanced post-menopausal breast cancer: clinical and endocrine effects

I.L. Crighton[1], M. Dowsett[2], A. Lal[2], A. Man[3] & I.E. Smith[1]

[1]*Department of Medicine, Royal Marsden Hospital, London SW3 6JJ;* [2]*Department of Biochemical Endocrinology, Royal Marsden Hospital, London SW3 6JJ; and* [3]*Lederle Laboratories, Gosport, Hampshire, UK.*

**Summary** Fifteen post-menopausal patients with advanced breast cancer were treated with the LH-RH agonist leuprorelin (D-leu$^6$-des-gly$^{10}$-Gn-RH-ethylamide) given in a dosage of 7.5 mg as a monthly subcutaneous depot injection, to assess the clinical activity and endocrine response to treatment. None of the 15 patients showed an objective response to treatment, although four patients had stable disease for at least 6 months. No toxicity was demonstrated. Endocrine effects after 4 weeks' treatment were as follows: mean levels of serum gonadotrophins fell to 10% of their pretreatment values; there were no significant changes in the levels of prolactin on treatment; there was a significant decrease in the levels of serum testosterone in 12 out of 14 patients; there were no significant changes in the levels of oestradiol, androstenedione and oestrone. The lowering of serum testosterone suggests that androgens in post-menopausal women may be partly produced by the ovaries, stimulated by LH and FSH. This fall in testosterone may explain why some post-menopausal breast cancer patients in other studies have been reported to respond to treatment with LH-RH agonists, as it would decrease the substrate for the peripheral synthesis of oestrogens.

Luteinising hormone-releasing hormone (LH-RH) agonists cause a reduction in the levels of plasma oestrogens in premenopausal women when given in a continuous, non-pulsatile manner. This is a result of down-regulation of pituitary receptors, which causes a decrease in the release of luteinising hormone, leading to a reduction in ovarian oestrogen synthesis (Furr & Milstead, 1988). Several of these agonists have been used in the treatment of premenopausal patients with metastatic cancer, with response rates of 30–40% (Klijn *et al.*, 1982; Harvey *et al.*, 1983; Nicholson *et al.*, 1985), similar to the results with oophorectomy or radiation-induced menopause (Stoll, 1979). More surprisingly, these agents have also been reputed to be effective in 16–20% of post-menopausal patients with advanced breast cancer (Harvey *et al.*, 1981; Plowman *et al.*, 1986). It has been suggested that response in this group may be due to a direct effect on the tumour, as some LH-RH agonists have been shown to have an inhibitory effect on breast cancer cells *in vitro* (Blankenstein *et al.*, 1985; Miller *et al.*, 1985), and LH-RH binding sites have been demonstrated in several breast cancer cell lines (Eidne *et al.*, 1987).

More recently, it has been shown that in post-menopausal breast cancer patients treated with an LH-RH agonist (goserelin), there was a significant reduction of serum testosterone levels, which was associated with a 22% fall in the level of serum oestradiol (Dowsett *et al.*, 1988). It is therefore possible that the response of post-menopausal patients to treatment with LH-RH analogues may be due to decreased oestrogenic stimulation, rather than to a direct, inhibitory effect on the tumour.

In this study, the clinical and endocrine response of 15 post-menopausal patients with advanced breast cancer was evaluated during their treatment with the LH-RH agonist leuprorelin (D-leu$^6$-des-gly$^{10}$-Gn-RH-ethylamide) (Lederle Laboratories, Gosport, Hampshire, UK).

### Patients and methods

Fifteen post-menopausal women with locally advanced or metastatic, histologically proven breast cancer were entered into the study. Informed consent was obtained from all patients. Their mean age was 66 ± 7.6 years (mean ± s.d.), range 49–77, the mean weight was 68.5 ± 15.8 kg, range 50.1–108.5, and all patients were at least 2 years post-menopausal. Two tumours were oestrogen receptor (ER) positive, one was ER negative, the receptor status of the remainder being unknown. The sites of disease are shown in Table I. After an initial assessment, which included physical examination, chest X-ray, isotope bone scan, liver ultrasound, electrocardiogram and urinalysis, treatment was commenced with 7.5 mg of leuprorelin given subcutaneously every fourth week. The leuprorelin was given as a depot injection, through a small gauge needle (23G), the drug being formulated as polylactic/polyglycolic acid microspheres. Blood samples for full blood count, platelets, urea and electrolytes, calcium, phosphate, liver function, and levels of gonadotrophins, oestrone, oestradiol, androstenedione and testosterone were taken pre-treatment, at weeks 1, 2 and 4, and thereafter just before each subsequent injection. Chest X-ray, bone and liver scans were repeated at 3-monthly intervals, and at suspected relapse. Treatment was discontinued when there was objective evidence of disease progression according to the WHO criteria of response (World Health Organization, 1979).

Five women had received no previous hormone therapy and 10 had previous treatment with tamoxifen, which in all cases had been stopped at least 4 weeks before commencing the leuprorelin therapy. No patients had received any other endocrine therapy. Two women had also previously received chemotherapy (melphalan and 5-fluoro-uracil, and methotrexate, mitoxantrone and mitomycin C). Of the women who had received therapy with tamoxifen, the mean length of time to progression was 17.7 ± 14.1 ( ± s.d.) months, with a range of 6–54 months. Three out of the 10 patients had shown an initial partial response to tamoxifen but had subsequently relapsed on treatment.

### Hormone measurements

Serum samples were stored at −20°C and assayed in batches for luteinising hormone (LH), follicle stimulating hormone (FSH), prolactin (Prl), oestradiol (E2), oestrone (E1), androstenedione ($\Delta^4$A) and testosterone (T) by radioimmunoassay techniques which have been described previously

**Table I** Disease sites in 15 post-menopausal breast cancer patients undergoing treatment with leuprorelin

| *Disease site* | *Number* |
|---|---|
| Breast | 9 |
| Soft tissue/nodes | 8 |
| Bone | 5 |
| Liver | 2 |
| Lung | 3 |

Correspondence: M. Dowsett.
Received 17 March 1989; and in revised form 3 May 1989.

(Ferguson *et al.*, 1982; Dowsett *et al.*, 1983, 1984, 1987*a*; Harris *et al.*, 1982, 1983). All samples from the same patient were analysed in the same batch. The intra- and inter-assay coefficients of variation were <10% and <15% respectively for all analytes.

*Statistical analyses*

The results were considered as percentages of pretreatment values, then a logarithmic transformation was taken on the data to normalise the distribution. The percentages were compared to 100% using paired *t* tests. The geometric mean percentages with 95% confidence limits are given in Figure 2. The geometric mean percentages with *P* values for week, 4, 8 and 12 are given in Table III. Multiple testing has been performed and a significance level lower than 5% should be considered. However, *P* values up to 10% are shown in Table III for extra information. The mean serum hormone levels before and after 4 weeks treatment are shown in Table IV.

### Results

*Clinical*

Response to treatment was determined according to standard WHO criteria for response. None of the patients showed an objective response to treatment. Four patients had stable disease for more than 24 weeks. One of these remains stable at 40 weeks. One patient who was disease stable at 24 weeks discontinued treatment with leuprorelin and had a course of radiotherapy to her localised breast carcinoma. The other two patients who initially had stable disease, showed progression after 24 weeks. Of the 13 patients who progressed on treatment (including the latter two), the mean time to progression was 15.8 ± 12.9 (± s.d.) weeks, range 2–40 weeks. Time to progression is demonstrated in Figure 1.

Subsequent treatment, and the best response to this is summarised in Table II.

**Figure 1** Actual time to progression in 14 post-menopausal breast cancer patients treated with leuprorelin. One patient was excluded who remained in stable disease at 6 months, and then had a course of radiotherapy.

**Table II** Treatment following therapy with leuprorelin, and the best response to this therapy

| *Response* | *MPA* | *Tamoxifen* | *Aromatase inhibitor* | *Chemotherapy* |
|---|---|---|---|---|
| PR | | 1 | 1 | 2 |
| NC | | 1 | 3 | 2 |
| PD | 1 | 1 | | |

*Toxicity*

None of the fifteen patients treated with leuprorelin reported any significant adverse reaction to the therapy and, in particular, there were no hot flushes or irritation at the site of injection. There were no observed haematological or biochemical abnormalities.

*Endocrine results*

The effect of leuprorelin on the levels of LH, FSH, Prl, T, $\Delta^4$A, E2, and E1 during the first 24 weeks of therapy are shown in Figure 2 as a percentage of the pretreatment value (geometric means and 95% confidence intervals). The arithmetic mean serum hormone levels before and after 4 weeks' treatment are shown in Table III. The most marked changes are seen in the levels of LH and FSH. LH levels fell to 63.4% of the pretreatment level at week 1, 28.5% at week 2 and 8.7% at week 4. Thereafter, they ranged between 6.2% and 10.2% of pretreatment levels. FSH fell more rapidly, to 30% of the pretreatment level at week 1, 10.3% at week 2 and 4.9% at week 4. Thereafter they varied between 5.8% and 14.5% of the pretreatment level. Five patients had pretreatment FSH levels which were less than 20 IU $l^{-1}$. However, all five patients were aged over 60 years, and these low levels probably reflect the decreased gonadotrophin secretion associated with advanced menopause.

Serum Prl levels showed no significant changes, although three individual patients showed marked increases in Prl levels to above 500 mIU $l^{-1}$ at the time of their disease progression.

Mean serum T levels fell significantly during treatment, to 74.1% of pretreatment levels at 4 weeks. Thereafter, the levels remained lower than the pretreatment levels at all time points. After 4 weeks' therapy, 12 out of the 14 patients on whom we had paired samples showed a lowering of their testerone levels (Figure 3), this being most marked in the patients whose pretreatment levels were greater than 1.5 nmol$^{-1}$. In these seven patients, levels fell from 2.09 ± 0.46 nmol $l^{-1}$ (mean ± s.d.) to 1.30 ± 0.43 nmol $l^{-1}$. In the seven patients with baseline levels less than 1.5 nmol $l^{-1}$, the levels fell from 1.25 ± 0.17 nmol $l^{-1}$ to 1.16 ± 0.27 nmol $l^{-1}$. In two patients, the serum testosterone levels showed a slight rise at 4 weeks. Mean $\Delta^4$A levels were also lower than the pretreatment levels for the first 4 weeks of therapy, but thereafter ranged between 87.3% and 132% of pretreatment levels.

E2 levels fell to 85.6% of the pretreatment value at week 4, and thereafter varied between 82.9% and 116% of pretreatment levels. In nine out of 14 patients, E2 levels fell after 4 weeks' therapy, in three patients levels increased and in two patients they remained unchanged (Figure 3). These changes were not as marked as the changes in the testosterone levels; statistical significance was approached only after 4 weeks. One patient had a pretreatment and on treatment level of >100 pmol $l^{-1}$. This patient was the heaviest patient in the study (108.5 kg), and also had the highest level of E1. Mean E1 levels fell to 85.0% of the pretreatment levels at 4 weeks, and thereafter ranged between 76.3% and 102.1% of the pretreatment levels.

### Discussion

In this study, none of our 15 patients showed an objective response to treatment with the LH-RH agonist, leuprorelin. This would correspond with a <5% chance of there being a >20% response rate. Thus our response rate was lower but not inconsistent with the earlier findings of Harvey *et al.* (1981), Plowman *et al.* (1986) and Harris *et al.* (1988a), who showed, respectively, a 16%, 20% and 11% partial response rate to therapy with an LH-RH agonist in post-menopausal breast cancer patients. In addition, in our study, four out of 15 patients had stable disease for at least 6 months, which may have been of value for those patients. While it would be

**Figure 2** Mean changes in levels of LH, FSH, T, $\Delta^4$A, E2 and E1 during leuprorelin therapy. Values are given as geometric means and 95% confidence limits. The number of patients was 15 on weeks 0, 1 and 2; 14 on week 4; 12 on week 8; 9 on week 12; 7 on week 16; 5 on week 20; 4 on week 24. The $P$ values at weeks 4, 8 and 12 are given in Table III.

**Table III** Geometric means of hormone levels, as a percentage of initial value, in post-menopausal patients with breast cancer at 4, 8 and 12 weeks after first treatment with leuprorelin

| *Hormone* | *Week 4* | *Week 8* | *Week 12* |
|---|---|---|---|
| LH | 8.7% $P<0.001$ ($n$ = 14) | 6.3% $P<0.001$ ($n$ = 12) | 7.0% $P<0.001$ ($n$ = 9) |
| FSH | 4.9% $P<0.001$ ($n$ = 14) | 5.8% $P<0.001$ ($n$ = 12) | 8.0% $P<0.001$ ($n$ = 9) |
| Prl | 123.5% n.s. ($n$ = 14) | 100.9% n.s. ($n$ = 12) | 123.1% n.s. ($n$ = 9) |
| T | 74.1% $P<0.002$ ($n$ = 14) | 79.9% $P<0.05$ ($n$ = 12) | 72.8% $P<0.005$ ($n$ = 9) |
| $\Delta^4$A | 96.4% n.s. ($n$ = 14) | 132.5% n.s. ($n$ = 12) | 101.8% n.s. ($n$ = 9) |
| E2 | 85.6% $0.05<P<0.10$ ($n$ = 14) | 96.9% n.s. ($n$ = 12) | 116.2% n.s. ($n$ = 9) |
| E1 | 85.0% $P<0.02$ ($n$ = 14) | 102.1% n.s. ($n$ = 12) | 99.9% n.s. ($n$ = 9) |

The percentages were compared to 100% using paired $t$ tests, after performing a logarithmic transformation.

biologically of interest to perform a larger study to define more precisely the response rate to these agents in post-menopausal patients this would be difficult to justify in circumstances where there are other clearly more effective agents from which to choose.

Endocrine measurements have confirmed that leuproreliin is a potent suppressor of LH and FSH levels, with a >90% fall in serum levels after 4 weeks, which was maintained for as long as therapy was continued. We have also demonstrated that leuprorelin causes a significant lowering of serum testosterone levels, particularly in those patients with pre-treatment levels of greater than 1.5 nmol $l^{-1}$. This confirms the findings of Dowsett *et al.* (1988) in goserelin-treated patients, and would support the suggestion that androgens in post-menopausal women may partly be produced by the ovaries, under the stimulation of pituitary gonadotrophins. There was a 15% drop in serum E2 levels after four weeks treatment, but this was of only borderline statistical significance ($0.05<P<0.10$), and was not sustained consistently, nor was any lowering of E2 levels associated with any lengthening of the time to progression of the disease. Although there are differences between the current study and that on goserelin treatment in the magnitude and statistical significance of the changes in plasma steroid hormone levels, the two studies are largely consistent in their indication of an ovarian suppressant effect of LHRH agonists in post-menopausal women which results in a relatively modest suppression of circulating androgen and oestrogen levels. These differences between the studies probably reflect the within-patient variability of the four steroids (Lonning *et al.*, 1989) and the between-patient variability in the endocrine response to LHRH agonist treatment.

One other interesting endocrine observation was that in three patients, serum prolactin levels rose markedly at the time of progression (from levels of 100, 110 and 130 mIU $l^{-1}$, to 1300, 560 and 670 mIU $l^{-1}$, respectively). This has previously been noted in patients progressing on other hormonal and cytotoxic treatments (Holtkamp *et al.*, 1984; Dowsett *et al.*, 1987*b*). It is probable that this is a result of, rather than a cause of, the disease progression.

In conclusion, leuprorelin in our hands was largely ineffective as a single agent therapy for the treatment of post-menopausal breast cancer. However, in 12 out of 14 patients, there was a decrease in the levels of serum testosterone, and, since androgens are a substrate for the peripheral production of oestrogens (Grodin *et al.*, 1973), it may be that combination endocrine therapy with an LH-RH agonist (to decrease the substrate for peripheral oestrogen synthesis), and an aromatase inhibitor (to suppress conversion of the substrate) would be more effective than treatment with an aromatase inhibitor alone.

We should like to thank Lederle for supplying the leuprorelin, and for funding the endocrine assays. We should also like to thank Sister D. Button and Miss D. MacKintosh for their help with the clinical data collection, and Norma Cherrington of Lederle for help with the statistical analyses.

**Figure 3** Individual changes in serum testosterone and serum oestradiol in 14 post-menopausal patients with breast cancer before (Base) and after 4 weeks' therapy with leuprorelin. The lines connect the values in the same patient.

**Table IV** Mean hormone levels in post-menopausal patients with breast cancer before and four weeks after first treatment with leuprorelin

| *Hormone* | *Pretreatment* (*n = 15*) | | *After 4 weeks* (*n = 14*) | |
|---|---|---|---|---|
| LH (IU $l^{-1}$) | 50.8 ± 16.2 | (5–210) | 2.7 ± 0.3 | (1.8–5.6) |
| FSH (IU $l^{-1}$) | 51.3 ± 10.8 | (10–160) | 3.5 ± 0.8 | (0.8–10) |
| Prl (mIU $l^{-1}$) | 143.3 ± 18.8 | (90–350) | 207.1 ± 51.5 | (90–730) |
| T (nmol $l^{-1}$) | 1.65 ± 0.14 | (1.0–2.8) | 1.23 ± 0.1 | (0.8–2.1) |
| $\Delta^4$A (nmol $l^{-1}$) | 2.57 ± 0.29 | (0.6–4.6) | 2.34 ± 0.29 | (0.9–5.1) |
| E2 (pmol $l^{-1}$) | 35.8 ± 8.3 | (12–130) | 34.2 ± 8.3 | (6–120) |
| E1 (pmol $l^{-1}$) | 162.7 ± 27.3 | (70–490) | 135.8 ± 18.5 | (50–310) |

Arithmetic mean ± s.e.m. (range).

### References

BLANKENSTEIN, M.A., HENKELMAN, M.S. & KLIJN, J.G.M. (1985). Direct inhibitory effect of a luteinizing hormone releasing hormone agonist on MCF-7 human breast cancer cells. *Eur. J. Cancer Clin. Oncol.*, **21**, 1493.

DOWSETT, M., McGARRICK, G.E., HARRIS, A.L. & 3 others (1983). Prognostic significance of serum prolactin levels in advanced breast cancer. *Br. J. Cancer*, **47**, 763.

DOWSETT, M., HARRIS, A.L., SMITH, I.E. & 1 other (1984). Endocrine changes associated with relapse in advanced breast cancer patients on aminoglutethimide therapy. *J. Clin. Endocrinol. Metab.*, **58**, 99.

DOWSETT, M., GOSS, P.E., POWLES, T.J. & 4 others (1987*a*). Use of the aromatase inhibitor 4-hydroxyandrostenedione in postmenopausal breast cancer: optimization of therapeutic dose and route. *Cancer Res.*, **47**, 1957.

DOWSETT, M., McGARRICK, G.E., HARRIS, A.L. & 3 others (1987*b*). The prognostic significance of hyperprolactinaemia. In *Hormonal Manipulation of Cancer: Peptides, Growth Factors, and New Anti-steroidal Agents*, Klijn, J.G.M. *et al.* (eds) p. 175. Raven Press: New York.

DOWSETT, M., CANTWELL, B., LAL, A. & 2 others (1988). Suppression of postmenopausal ovarian steroidogenesis with the luteinizing hormone releasing hormone agonist goserelin. *J. Clin. Endocrinol. Metab.*, **66**, 672.

EIDNE, K.A., FLANAGAN, C.A., HARRIS, N.S. & 1 other (1987). Gonadotrophin releasing hormone (Gn-RH) binding sites in human breast cancer cell lines and inhibitory effects of Gn-RH antagonists. *J. Clin. Endocrinol. Metab.*, **64**, 425.

FERGUSON, K.M., HAYES, M. & JEFFCOATE, S.L. (1982). A standardized multi-centre procedure for plasma gonadotrophin radioimmunoassay. *Ann. Clin. Biochem.*, **19**, 358.

FURR, B.J.A., MILSTEAD, R.A.V. (1988). LH-RH analogues in cancer treatment. In *Endocrine Management of Cancer: 2: Contemporary Therapy*, Stoll, B.A. (ed) p. 16. S. Karger AG: Basel.

GRODIN, J.M., SIITERI, P.K. & MACDONALD, P.C. (1973). Sources of oestrogen production in postmenopausal women. *J. Clin. Endocrinol. Metab.*, **36**, 207.

HARRIS, A.L., CARMICHAEL, J., CANTWELL, B.M.J. & DOWSETT, M. (1989). Zoladex: endocrine and therapeutic effects of postmenopausal breast cancer. *Br. J. Cancer*, **59**, 97.

HARRIS, A.L., DOWSETT, M., JEFFCOATE, S.L. & 3 others (1982). Endocrine and therapeutic effects of aminoglutethimide in premenopausal patients with breast cancer. *J. Clin. Endocrinol. Metab.*, **55**, 718.

HARRIS, A.L., DOWSETT, M., JEFFCOATE, S.L. & 1 other (1983). Aminoglutethimide dose and hormone suppression in advanced breast cancer. *Eur. J. Cancer Clin. Oncol.*, **19**, 493.

HARVEY, H.A., LIPTON, A., SANTEN, R.J. & 7 others (1981). Phase II study of a gonadotrophin releasing hormone analog, leuprolide, on postmenopausal advanced breast cancer patients. *Proc. Am. Assoc. Cancer Res. Am. Soc. Clin. Oncol.*, **22**, 444 (abstract C-436).

HARVEY, H.A., LIPTON, A. & MAX, D.T. (1983). Medical castration produced by the Gn-RH analogue leuprolide to treat metastatic breast cancer. *J. Clin. Oncol.*, **3**, 1068.

HOLTKAMP, W., NAGEL, G.A., WANDER, H.-E. & 1 other (1984). Hyperprolactinaemia is an indicator of progressive disease and poor prognosis in advanced breast cancer. *Int. J. Cancer*, **34**, 323.

KLIJN, J.G.M. & DE JONG, F.H. (1982). Treatment with a luteinizing hormone releasing hormone (Buserelin) in premenopausal patients with metastatic breast cancer. *Lancet*, **ii**, 1213.

LONNING, P.E., DOWSETT, M., SCHEM, B., HARDY, J., JACOBS, S. & POWLES, T.J. (1989). Lack of diurnal variation in plasma oestradiol, oestrone, androstenedione and testosterone in postmenopausal women. *J. Steroid Biochem.* (in the press).

MILLER, W.R., SCOTT, W.N., MORRIS, R. & 2 others (1985). Growth of human breast cancer cells inhibited by a luteinizing hormone releasing hormone agonist. *Nature*, **313**, 2231.

NICHOLSON, R.I., WALKER, K.J., TURKES, A. & 4 others (1985). Endocrinological and clinical aspects of LH-RH action (ICI 118630) in hormone dependent breast cancer. *J. Steroid Biochem.*, **23**, 843.

PLOWMAN, P.N., NICHOLSON, R.I. & WALKER, K.J. (1986). Remission of postmenopausal breast cancer during treatment with the luteinising hormone releasing hormone agonist ICI 118630. *Br. J. Cancer*, **54**, 903.

STOLL, B.A. (1972). Castration and oestrogen therapy. In *Endocrine Therapy in Malignant Disease*, Stoll, B.A.(ed), p. 139. W.B. Saunders: Philadelphia.

WORLD HEALTH ORGANIZATION (1979). *Handbook for Reporting the Results of Cancer Treatment*. WHO: Geneva.